# Ranking antibody binding epitopes and proteins across samples from whole proteome tiled linear peptides

**DOI:** 10.1093/bioinformatics/btae637

**Published:** 2024-11-05

**Authors:** Sean J McIlwain, Anna Hoefges, Amy K Erbe, Paul M Sondel, Irene M Ong

**Affiliations:** Department of Biostatistics and Medical Informatics, University of Wisconsin-Madison, Madison, WI, 53705, USA; University of Wisconsin Carbone Comprehensive Cancer Center, University of Wisconsin-Madison, Madison, WI, 53705, USA; Department of Human Oncology, University of Wisconsin-Madison, Madison, WI, 53705, USA; Department of Human Oncology, University of Wisconsin-Madison, Madison, WI, 53705, USA; University of Wisconsin Carbone Comprehensive Cancer Center, University of Wisconsin-Madison, Madison, WI, 53705, USA; Department of Human Oncology, University of Wisconsin-Madison, Madison, WI, 53705, USA; Department of Pediatrics, University of Wisconsin-Madison, Madison, WI, 53705, USA; Department of Biostatistics and Medical Informatics, University of Wisconsin-Madison, Madison, WI, 53705, USA; University of Wisconsin Carbone Comprehensive Cancer Center, University of Wisconsin-Madison, Madison, WI, 53705, USA; Department of Obstetrics and Gynecology, University of Wisconsin-Madison, Madison, WI, 53705, USA; Center for Human Genomics and Precision Medicine, University of Wisconsin-Madison, Madison, WI, 53705, USA

## Abstract

**Introduction:**

Ultradense peptide binding arrays that can probe millions of linear peptides comprising the entire proteomes of human or mouse, or hundreds of thousands of microbes, are powerful tools for studying the antibody repertoire in serum samples to understand adaptive immune responses.

**Motivation:**

There are few tools for exploring high-dimensional, significant and reproducible antibody targets for ultradense peptide binding arrays at the linear peptide, epitope (grouping of adjacent peptides), and protein level across multiple samples/subjects (i.e. epitope spread or immunogenic regions of proteins) for understanding the heterogeneity of immune responses.

**Results:**

We developed **H**ierarchical antibody binding **E**pitopes and p**RO**teins from li**N**ear peptides (HERON), an R package, which can identify immunogenic epitopes, using meta-analyses and spatial clustering techniques to explore antibody targets at various resolution and confidence levels, that can be found consistently across a specified number of samples through the entire proteome to study antibody responses for diagnostics or treatment. Our approach estimates significance values at the linear peptide (probe), epitope, and protein level to identify top candidates for validation. We tested the performance of predictions on all three levels using correlation between technical replicates and comparison of epitope calls on two datasets, and results showed HERON’s competitiveness in estimating false discovery rates and finding general and sample-level regions of interest for antibody binding.

**Availability and implementation:**

The HERON R package is available at Bioconductor https://bioconductor.org/packages/release/bioc/html/HERON.html.

## 1 Introduction

The technology for high-dimensional identification of specific antibody binding repertoire has significantly improved over the last decade, allowing for the determination of antibody binding to ∼6 million peptides simultaneously with peptide array technology to probe every mouse or human protein using 16-mer peptides with 1, 2, or 4 amino acid (a.a.) tiling to identify antibody targets from serum or plasma (Lyamichev *et al.* 2017). This extremely high-dimensional, high-throughput method for antigen-specific immune profiling can enable detection of immune responses to infection or vaccines, and study the precise targeting of tumors by one’s own immune system. There are many existing methods for analyzing microarray and peptide array data (Nahtman *et al.* 2007, Flinterman *et al.* 2008, Lin *et al.* 2009, Renard *et al.* 2011, Lin *et al.* 2012, Imholte and Gottardo 2016). PepStat (Imholte *et al.* 2013) was designed to compare antibody binding to different viral strains for a single protein, and a few methods were developed for analyzing these ultra-dense, high-dimensional array data (Heffron *et al.* 2018, Lo *et al.* 2020, Heffron *et al.* 2021, Zheng *et al.* 2021, Chen *et al.* 2022, Mergaert *et al.* 2022, Potluri *et al.* 2022), however, additional methods are necessary to advance the understanding of immune response. Antibodies may bind to peptides via various mechanisms of interaction based on the antibody’s antigen binding site and amino acid configuration of the peptides, affecting binding affinity, and each protein may have multiple epitopes that are bound by antibodies, amplifying the immune response to the protein. These considerations are important when studying immune responses involved in diseases, whether infectious, auto-immune, or neoplastic.

Existing methods for analyzing antibody binding of peptide array data such as pepStat (Imholte *et al.* 2013), estimates the false discovery rate (FDR) at a peptide-level and reports subject-level statistics. One Bayesian model, pepBayes, has been shown to be potentially superior to pepStat, however, it was also designed to analyze a single protein from multiple related viral strains and does not take into account the adjacent probes across the protein (Imholte and Gottardo 2016). Another Bayesian approach includes provision for handling sequential probe signals by using a latent autoregressive component, however, the authors propose a limit in the problem size (300 peptides and 50 samples) when using their implementation (Arima *et al.* 2012). MixTwice (Zheng *et al.* 2021) is a statistical method that utilizes additional power to detect significant probes by using local FDR.

To our best knowledge, there are currently no existing method that can simultaneously identify and rank antibody binding responses at different scales, i.e. to linear peptides, epitopes (defined here as antibody bound region of adjacent linear peptides or probes tiled within a protein) and proteins, for diagnostics or treatment; or for understanding the heterogeneity of immune response within an individual or across a population while scaling to handle whole proteome peptide arrays with ∼6 million unique sequence probes.

We developed an algorithm, HERON (**H**ierarchical antibody binding **E**pitopes and p**RO**teins from li**N**ear peptides), available on Bioconductor, for analyzing ultra-dense peptide arrays to identify and rank significantly bound linear peptides, epitopes, and proteins. Our approach builds on existing approaches (Imholte *et al.* 2013) including clustering methods to locate contiguous probes (i.e. epitopes) with high binding affinity, and meta-analysis methods from Fisher and others (Wilkinson 1951, Becker 1994, Poole *et al.* 2016, Wilson 2019, Liu and Xie 2020, Dewey 2022) to: (i) allow for reliability and reproducibility with granularity in confidence level for making antibody binding calls at different thresholds, (ii) ensure that we identify probes that are more highly bound in positive (experimental or post-treatment) samples compared to negative samples (control or pre-treatment), and that we give more weight to those with higher overall binding, (iii) identify consecutive overlapping probes with high binding signal and categorize the shared amino acid (a.a.) sequences represented by those highly recognized probes as epitopes based on specified thresholds, and (iv) identify proteins due to epitope spread (i.e. proteins recognized by distinct epitope binding in different regions of the protein by sera from multiple samples, not necessarily at the same place on the protein). The performance is evaluated on two datasets to illustrate its utility for processing and analyzing peptide binding array data. The goal of the first dataset (COVID-19) was to identify diagnostic epitopes across the landscape of the SARS-CoV-2 proteome as detected in human serum samples from individuals following SARS-CoV-2 infection (Heffron *et al.* 2021). The goal of the second dataset (Melanoma) was to study the landscape of antibody responses to the whole mouse proteome of genetically identical mice before cancer and after curative immunotherapeutic *in situ* vaccine treatment (Morris *et al.* 2016) and rechallenge with a related tumor and to rank the responses for study and validation.

## 2 Materials and methods

### 2.1 Datasets

The COVID-19 dataset (Heffron *et al.* 2021) compares analyses of serum samples from individuals following proven infection with SARS-CoV-2 (COVID+) with serum samples from uninfected individuals (COVID-) to identify diagnostic epitopes across the landscape of the SARS-CoV-2 proteome; this dataset was downloaded from https://github.com/Ong-Research/UW_Adult_Covid-19. There are 60 (20 COVID-, 40 COVID+) samples in total, with 118 651 unique sequence probes mapped to 470 086 16-mer probes for 387 proteins with a tiling of 1 amino acid (a.a.) and up to 5 replicates on the array. The downloaded data that had already been processed using pepMeld, https://github.com/DABAKER165/pepMeld (Baker 2020) was quantile normalized at the sequence probe level.

The Melanoma dataset (Hoefges *et al.* 2023) aims to study the landscape of antibody responses to the whole mouse proteome of genetically identical mice before cancer and after curative immunotherapeutic *in situ* vaccine treatment (Morris *et al.* 2016) and rechallenge with a related tumor and to rank the responses for study and validation. The dataset, available at Zenodo (https://doi.org/10.5281/zenodo.7871565), consists of 13 biological (serum) samples tested for recognition of 6 090 593 16-mer unique sequence probes mapped to 8 459 970 protein probes using a mixed tiling of either 2 a.a. or 4 a.a. with a total of 53 640 individual proteins. There are five naïve mice samples (referred to as “negative”; i.e. prior to tumor introduction or treatment), and eight immune samples (referred to as “positive”; i.e. tumor-bearing mice that underwent radiation and immunotherapy treatment to become tumor-free and were subsequently rechallenged with the tumor line to test immune memory). The data from Zenodo had already been pre-processed by Nimble Therapeutics, quantile normalized, and then smoothed, hence we applied our analyses to the downloaded data without further preprocessing.

### 2.2 Algorithm

**Figure 1.**
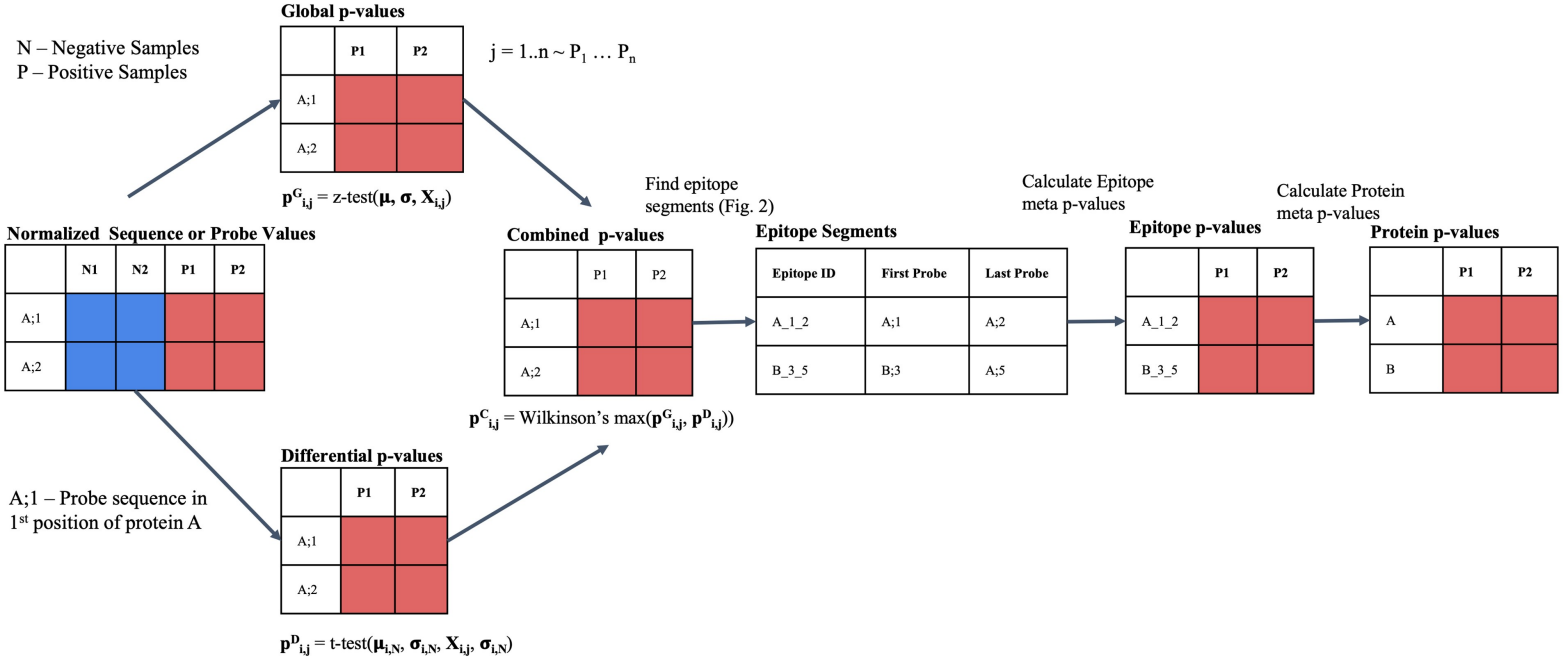
HERON workflow for processing peptide binding array data. Workflow illustrating algorithm that takes as input a normalized matrix of sequence probes and samples and outputs probe-level, epitope-level, and protein-level differential antibody binding calls on each sample. The combined sequence *P*-value are calculated by combining a global *P*-value from a one-sided *z*-test with a differential *P*-value from a one-sided *t*-test using Wilkinson’s max. The N1 and N2 columns indicate the negative sample values and output and the P1 and P2 columns indicate the positive sample values and output. After adjustment of the sequence *P*-values using Benjamini–Hochberg, the raw sequence *P*-values and adjusted sequence *P*-value are copied to the respective protein probe locations. After correction using BH, an FDR cutoff is used to identify the probes significantly differentially bound by antibodies. The epitope regions, where the Epitope ID is defined by Protein_FirstProbe_LastProbe, are detected using a segmentation algorithm, and the found segments are scored using meta-analyses methods to combine the probe-level *P*-values, corrected using BH, and an FDR threshold is used to identify significant differentially bound antibodies. Finally, the protein scores are determined from the associated protein’s epitope *P*-value(s) using meta-analyses methods, corrected using BH, and then called using an FDR threshold.

HERON is a comprehensive R package for analyzing ultra-dense peptide binding arrays with multiple protein sequences, e.g. whole mouse or human proteomes, to rank or calculate a *P*-value/false discovery rate (FDR) for each linear peptide probe for each positive sample and summarize ranking at different resolutions (including epitope and protein levels) for multiple positive samples available at Bioconductor (https://bioconductor.org/packages/release/bioc/html/HERON.html). The algorithm workflow is illustrated in Fig. 1.

#### 2.2.1 Estimating probe-level *P*-values for each positive sample

We first calculate a global *P*-value using a distribution function of all the data provided in the experiment. Next, we calculate a differential *P*-value using a one-sided *t*-test for each positive sample assuming that the standard deviation is the same between groups using the negative or positive samples. Parameters for generating probe-level *P*-values can be adjusted and one can use either or both global and differential tests when calculating significance for peptides to identify probes that are more highly bound in positive samples compared to the negative samples or give more weight to those with higher overall binding, respectively. Our approach calculates a combined *P*-value from the global and differential *P*-value results using the Wilkinson's max meta *P*-value method (Tippett 1931, Wilkinson 1951, Birnbaum 1954, Becker 1994). The linear probe-level *P*-values are then adjusted for false discovery rates (FDR) using the Benjamini-Hochberg algorithm. Supplementary Figure S1 shows the estimated global *P*-values (S1A), differential *P*-values (S1B), combined global and differential *P*-values (S1C), and adjusted *P*-values (Benjamini-Hochberg, S1D) versus the peptide array signal after applying the HERON workflow on data for a positive sample from the Melanoma dataset. An FDR threshold cutoff is used to identify the set of antibody-bound probes (which we will also refer to as “calls” or “called” probes) for each positive sample.

#### 2.2.2 K of N calls and one-hit filter

HERON reports the number of samples that have an adjusted *P*-value less than the specified threshold, i.e. K out of N (or %Called) that indicate antibody binding for the called probes. To filter out inconsistent calls due to spurious noise (or nonspecific signals), the algorithm can employ a one-hit filter for removing called probes that do not have a supporting consecutive probe call in the same sample nor a call in another serum sample. The one-hit filtering is employed on the called probes and the respective adjusted *P*-values are set to 1. These procedures are also applied to epitope- and protein-level probes after the steps described below.

#### 2.2.3 Epitope finding

Using the set of identified antibody-bound probes, epitopes can be identified using one of three different segmentation algorithms: unique, hierarchical, or skater.

**Figure 2.**
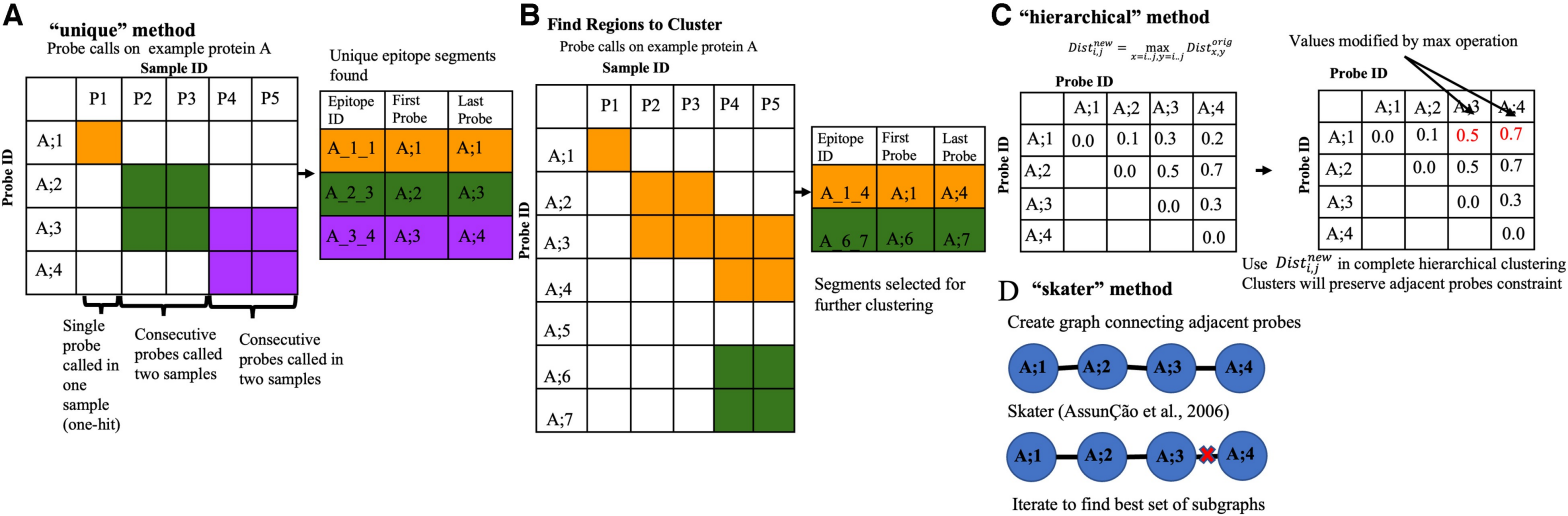
Illustration and example of epitope finding algorithms implemented in HERON. (A) Illustration of the “unique” method, which finds the unique set of grouped probes called from each positive sample. The cells highlighted in orange, green, or purple are the called probes. The table to the right of the illustration is filled in with the color corresponding to the outlined boxes in the illustration (orange, green, and purple) to indicate the called epitope probe regions in the illustration. Epitope ID is defined by Protein_FirstProbe_LastProbe of the epitope. (B) Illustration of how regions are found to further cluster using either the hierarchical or skater method. The orange and green highlighted boxes mark the probes that are adjacent and the table on the right indicates the larger groups of probes, in the corresponding orange and green, that will be further segmented using the clustering methods. (C) Illustration of the hierarchical clustering method, which adapts hierarchical clustering to find consecutive probes with consistent call patterns across samples. The matrix on the left is the original distance matrix calculated from the dissimilarity of the probe calls or scores. The matrix on the right is the new matrix after applying the max operation. The two red numbers indicate the cells that were changed due to the max operation. (D) Illustration of the “skater” method which decomposes a linear graph of consecutive probes to find consistent call patterns across samples.

The “unique” method iterates through all the samples, finding consecutive runs of probes that were called on the same protein, then combines all the regions found across the samples into a single list, and reports the set of blocks/epitopes, which includes overlapping blocks. Figure 2A illustrates the “unique” algorithm on an example set of called probes.

The clustering approaches (hierarchical and skater) first finds regions within each protein where consecutive probes are called in any positive sample (Fig. 2B), then the hierarchical (hclust; Murtagh 1985; Fig. 2C) or skater (spdep; Bivand *et al.* 2013, Bivand and Wong 2018; Fig. 2D) clustering method with the average silhouette score as threshold is used to determine the optimal number of clusters or graph cuts respectively.

To calculate similarity or dissimilarity between probes in relation to their significance scores or calls for the post samples, HERON provides the option of a “binary” or a “*z*-score” approach. The binary method uses the probe calls as labels (true/false for bound/unbound by an individual serum sample) for which a hamming distance can be calculated between the probes. The z-score method converts the probe *P*-values to a one-sided z-score before computing the Euclidean distance between probes. For both the hierarchical and skater segmentation implementations, the maximum average silhouette score was used to determine the number of clusters or cuts respectively. Ties in the silhouette score are broken by selecting the results with the maximum number of clusters or cuts.

For the hierarchical clustering approach, for each identified cluster, a distance matrix is derived by setting each *i*/*j* element to the maximum pairwise distance between probes starting at protein position *i* and ending at protein position *j* for the group of probes to be segmented. Complete hierarchical clustering is then performed using the distance matrix to find clusters that are contiguous probes within the protein (Fig. 2C).

The skater clustering approach utilizes graph constraints for the clustering process by defining a linear graph where an edge is introduced between every adjacent peptide within the protein tiling. Each iteration of the skater algorithm finds the best cut in the graph using the calculated distance metric between the probes. After iterating through all possible cuts to find the cut that gives the best average silhouette score, the epitope regions are then defined by the remaining connected nodes in the graph (Fig. 2D).

#### 2.2.4 Epitope and protein *P*-values

An epitope level *P*-value is calculated by using a meta *P*-value method with each positive sample’s linear probe *P*-values for the probes that are contained in that epitope. Similarly, the protein level *P*-value is determined using a meta *P*-value method based on the epitope *P*-values found across the protein for each positive sample. Many meta *P*-value estimation methods exist (Wilkinson 1951, Becker 1994, Alves and Yu 2014, Poole *et al.* 2016, Wilson 2019, Huo *et al.* 2020, Liu and Xie 2020, Sun and Lin 2020), including Fisher, and each has its own characteristics depending upon the type of underlying hypothesis to be tested.

For epitopes, we chose a meta *P*-value method that requires most, if not all, of the peptide probes within the epitope block to have significant values, such as Wilkinson’s max (wmax) (Tippett 1931, Wilkinson 1951, Birnbaum 1954, Becker 1994). To relax the requirement that all probe *P*-values within an epitope be significant, Wilkinson’s max can be calculated on the *n*^th^ maximum *P*-value (i.e. wmax2 for the 2^nd^ highest *P*-value within the epitope). In cases where Wilkinson’s max is used and the number of probes in the epitope is fewer than n peptides, HERON conservatively sets the *P*-value to 1. Due to the inherent possibility of dependency between the signals of adjacent probes, options such as the harmonic mean (hmp; Wilson 2019) and the Cauchy Combination Test (cct; Liu and Xie 2020) meta *P*-value methods, which are more tolerant to inter-dependencies between the elements for which the *P*-values are combined are also provided as options in HERON.

At the protein level, the goal is to identify proteins where at least 1 or more epitopes are significant, thus a meta *P*-value method of choice would be the minimum + Bonferroni correction (min_bonf) or the Wilkinson’s minimum (wmin) or Tippett’s method (Tippett 1931, Wilkinson 1951, Birnbaum 1954, Becker 1994). Using the *n*^th^ minimum (i.e. wmin2 for the 2^nd^ smallest epitope *P*-value within the protein) would be a more stringent requirement, where at least *n* epitopes must be significant. In the case where there are fewer than n epitopes with *P*-values, HERON utilizes Wilkinson’s min with the (*n*−1)^th^*P*-value iteratively. If there is only one *P*-value, then Wilkinson’s *n*^th^ min will just return the single *P*-value.

## 3 Results

The performance of the algorithm and optimal parameter settings were tested on two different datasets, COVID-19 and Melanoma. Performance was evaluated by testing the correlation between technical replicates for repeatability of calls, finding peptides that ensure reproducibility with a validation assay such as ELISA, and epitope boundary finding parameters as described in the next sections.

### 3.1 Performance on COVID-19 dataset

We compared HERON’s performance on a COVID-19 dataset, using parameters that were similar to the *t*-test workflow used to identify diagnostic peptides (Heffron *et al.* 2021). We utilized the following significance parameters: an absolute shift on the differential *t*-test of one, where one would mean the difference between the current COVID+ sample’s normalized fluorescent value from the Nimble system sample and the average normalized values from the COVID− samples is significantly different by more than 2-fold, an adjusted *P*-value threshold <0.01, using one-hit filter, hierarchical clustering with binary and hamming distance scores, Williamson’s max and Tippett’s for epitope and protein meta *P*-value estimations respectively. Probe, Epitope, and Protein calls were made if 25% of the COVID+ samples were called at the adjusted *P*-value threshold of <0.01. We use just HERON’s differential *t*-test to resemble the processing done in the Heffron *et al.* (2021) paper. To compare against other methods, we ran pepStat using its normalization method, without smoothing, and an FDR cutoff of 0.01.

**Figure 3.**
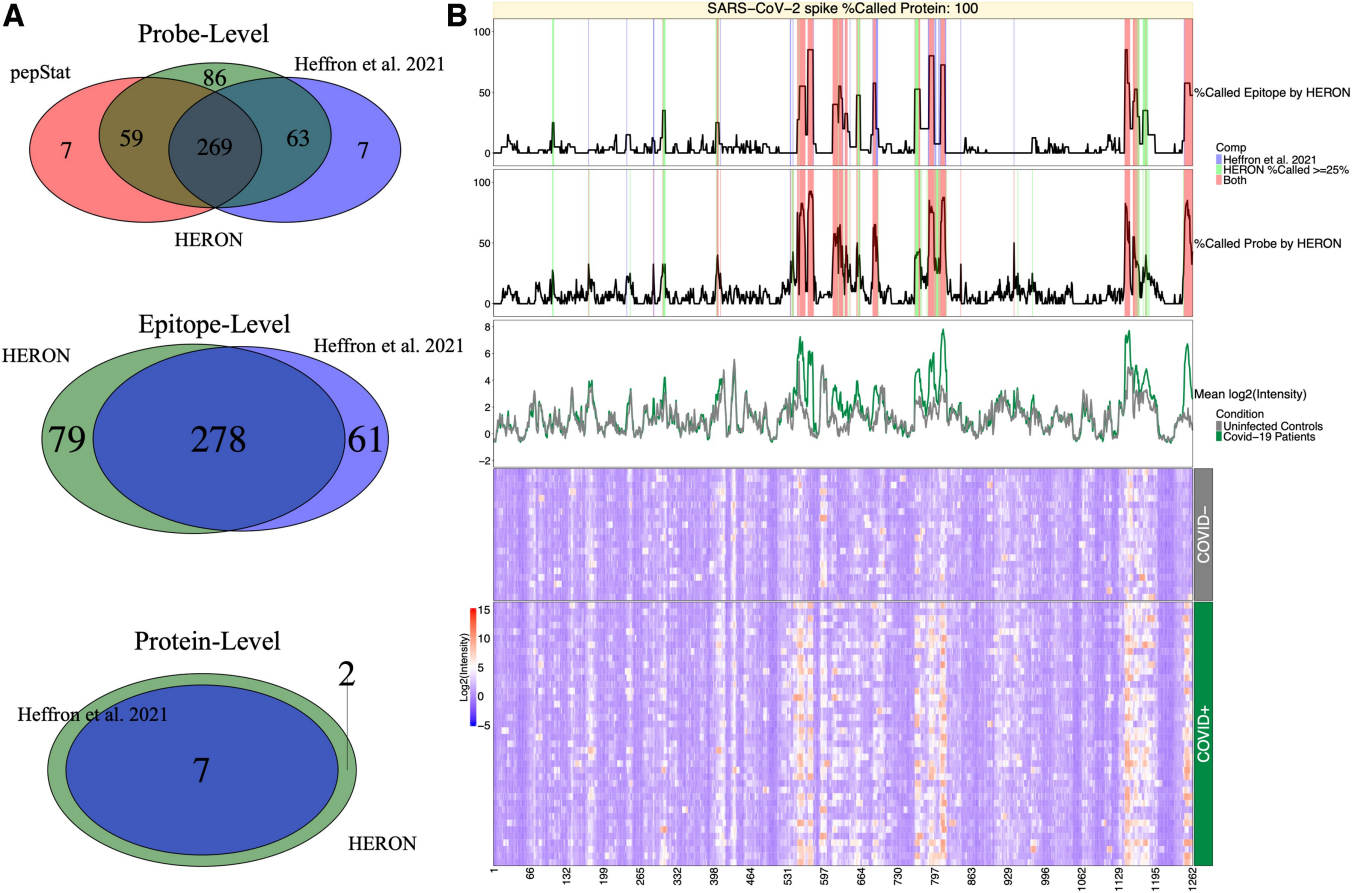
Venn diagram comparisons for COVID-19 dataset and Heatmap of SARS CoV-2 spike protein. (A) Venn diagrams of the linear peptide probe level (top), epitope level (middle), and protein level (bottom) where the green circle represents the calls made by HERON, the red circle represents the probe calls made by pepStat (Imholte *et al*. 2013), and the blue circle represents the calls made by Heffron *et al*. (2021). (B) Annotated heatmap (Gu *et al*. 2016, Gu 2022) of SARS CoV-2 spike protein. The heatmap (at the bottom) depicts the normalized intensity values for the probes (*x*-axis) tiled across the SARS-CoV-2 membrane protein and the individual patient serum samples (*y*-axis) with red representing high antibody (Ab) binding and blue representing low Ab binding. The top line plot indicates the percent of COVID-19 positive (COVID+) samples that were called as significantly differentially bound at least 2-fold over the average of the COVID-19 negative (COVID-) samples and after calculating the meta *P*-values (BH corrected, *P*-adj < 0.01) on the epitope level, the middle line plot indicates the percent of COVID+ samples called significantly 2-fold over the COVID-samples on the probe level (*P*-adj < 0.01), and the bottom line plot shows the average signal between control (COVID-) and positive samples. In these line plots, the % Called Probe by HERON and % Called Epitope by HERON are highlighted in red if both the HERON and Heffron *et al*. (2021) method called the same probes or group of probes, blue if only the Heffron *et al*. (2021) method called the probe or epitope, and light green if the probe or epitope was called by only the HERON method.

Each segmentation result and subsequent calls at the probe, epitope and protein levels are compared against the previously reported regions (Heffron *et al.* 2021; Fig. 3A). For the epitope level, we convert the selected epitopes back to the list of probes that are contained within each epitope, and then compare the probes between the two methods. Most of the probes (332 of 339, 97.9%) that were previously identified by Heffron *et al.*, were also identified by HERON indicating good agreement. Seven of the probes missed by HERON are part of short (1–2 probes) epitopes. Conversely, of the 145 probes identified in HERON but not in Heffron *et al.*, 56 just missed being called (exactly 10 out of 40 COVID+ samples). The overlaps and probes unique to Heffron *et al.* (2021) and the HERON method are provided in Supplementary Table S1. We also compared HERON’s *t*-test method against a Wilcoxon implementation within HERON and with the calls found using pepBayes using the same cutoffs (Supplementary Fig. S2). We observed that the probe-level hits in the Wilcoxon are quite similar in the amount and content of hits found, while pepBayes calls upon a subset of the probes found by the Wilcoxon and *t*-test methods.

Figure 3B shows a heatmap of antibody binding to the spike protein with line graphs showing the probe and epitope annotations of the SARS-CoV-2 spike protein from the heat map displayed across the top. The epitope and probe annotations also display the agreements and disagreements between HERON and the epitopes and probes as previously reported (Heffron *et al.* 2021). The differences between the epitope calls are most likely due to the difference in the method for finding epitope segments and the meta *P*-value method used to score them. The overlaps and epitope probes unique to the Heffron or HERON methods are provided in Supplementary Table S2. Of the 61 epitope probes that were missed by HERON, 43 are due to an epitope extension, where HERON finds a longer epitope than the previous method, but results in a loss of significance. 3 of the 43 extensions are close to the of 25% threshold (9/40 = 22.5%) for called COVID+ samples. The remaining 18 have matching epitopes in HERON but are shifted or fragmented and have a loss of significance. Subsequently, of the 79 epitope probes found in HERON but not in the previous method, 49 are extensions (with 9 on the edge, i.e. 10 out of 40 COVID+ samples called), and 21 are from new epitopes (with 6 of the 21 on the edge of being called).

The protein level Venn diagram at the bottom in Fig. 3A shows HERON identified more proteins than the previous method (Heffron *et al.* 2021). Since the requirement on the protein level is to have one or more significant epitopes and many potential epitopes are detected on the proteins, the proteins selected in the COVID dataset will be permissive in the number of proteins called.

### 3.2 Repeatable significance values in melanoma dataset

Technical replicate samples from the murine Melanoma immunotherapy dataset were used to study repeatable significance values: one single immune serum sample was divided into two identical aliquots which were tested in parallel with parallel datasets collected in the same array assay (B2), or a separate single immune serum sample divided into two identical aliquots which were tested on similar arrays, independently with parallel datasets collected in two separate similar arrays that were performed approximately one year apart (PD1).

For the parameters specific to probe *P*-value calculations, we chose three different levels of significance on the global z-test *P*-value coupled with the FDR cutoff used [Inclusive: global with a standard deviation (SD) shift of 3 and adjusted *P*-value cutoff of <0.2, Moderate: global sd shift of 6 and adjusted *P*-value cutoff of <0.05, and Restrictive: global sd shift of 10 and adjusted *P*-value cutoff of <0.01] and investigated with or without the use of the one-hit filter. For the epitope *P*-value parameters, we chose five different ways of finding epitopes [unique, hierarchical clustering with binary calls and hamming distance (hbh) or z-score with Euclidean distance (hze), skater with binary calls and hamming distance (sbh), and skater with z-score and Euclidean distance (sbz)]. The epitope meta *P*-value was either Wilkinson’s max (wmax1), Wilkinson’s 2^nd^ max (wmax2), Fisher (fisher), harmonic mean (hmp), or Cauchy (cct). For protein meta *P*-values, we choose either Fisher, Wilkinson’s min (wmin1), Wilkinson’s 2^nd^ min (wmin2), or min with a Bonferroni correction (min_bonf). Different adjusted *P*-value cutoffs could be used for the probe, epitope, and protein levels. Our results have tied these three parameters to the same value.

We calculated the Pearson correlation between the technical replicates of B2 and PD1 using the -log10(adjusted *P*-values) for the probes, epitopes, and proteins using an exhaustive search of the parameters mentioned above. The correlation results are presented in Supplementary Table S3.

Looking at the average correlations between the probe-level *P*-values of the technical replicates of B2 and PD1 (Supplementary Table S4) and marginalizing across the one-hit filter results, we found that using more stringent statistical filtering improves the technical replicate Pearson intercorrelation of the probe –log_10_ (adjusted *P*-values) (Inclusive: 0.806, Moderate: 0.841, and Restrictive: 0.846). By marginalizing across the statistical parameters, we also found that using the one-hit filter slightly improves the average replicate intercorrelation (Without: 0.824, With: 0.837). To find a good setting with good repeatability across the probe level significance values used, we then averaged the average correlation for the probe, epitope, and protein across the inclusive, moderate, and restrictive statistical parameters. The overall correlation results are reported in Supplementary Table S5 and the top 10 parameter settings are summarized in Supplementary Fig. S3A. Looking at the top 10, it appears that the Wilkinson's max on the 2^nd^ highest *P*-value for epitopes and either Tippett’s or min+Bonferroni for protein meta *P*-values using the skater or hierarchical clustering segmentation methods on the binary calls achieve the highest average correlation across the significance levels and the two technical replicates.

The choice of segmentation method can determine the length of the epitopes (Supplementary Fig. S3B). It appears, on average, that the “unique” (uniq) segmentation method gives longer epitopes, while the skater or hclust segmentation methods using the binary calls results in shorter epitopes. Evaluating results in Supplementary Fig. S3A, the shorter epitopes may indeed be more accurate since the skater or hclust methods are within the top 2 parameters for highest average technical correlation.

Supplementary Figure S4 show technical replicates as scatterplots for each of the probe, epitope, and proteins for the best overall average correlation using the Moderate global standard deviation shift and FDR cutoff values.

The increase of correlation from probes to epitope to proteins respectively can be attributed to the smaller number of entities to calculate the correlation from and the recruitment of correlated probes within the epitope, which is similar to averaging across correlated signals.

### 3.3 Probe, epitope, and protein counts

**Figure 4.**
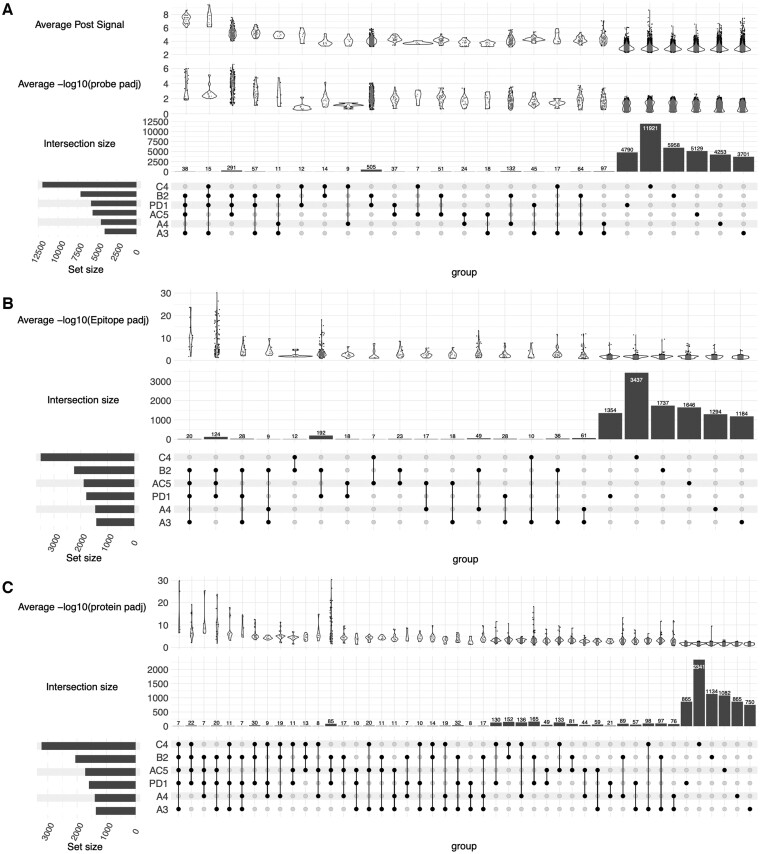
Upset Plots with Average Post Signal and −log10 adjusted *P*-values. Upset plot of the probes (A), epitopes (B), and proteins (C) called for each sample using the moderate significance level (min set size = *X*, sorted by the decreasing number of categories in the intersection set). AC5, A3, C4, A4, B2, and PD1 are the designations for the individual mice cured of melanoma, that provided the six separate immune serum samples tested here. For the probe plot (A), the top row of violin plots shows the average post signal for each intersection, the middle row of violin plots shows the average of −log_10_(probe adjusted *P*-values) for each intersection. The epitope (B) and protein (C) violin plots are the respective −log_10_(epitope adjusted *P*-value) and −log10(protein adjusted *P*-values).

We used the best correlation parameters to make calls on the Melanoma dataset for which the technical replicates were averaged together at the inclusive, moderate, or restrictive significance levels to obtain calls at the probe, epitope, and protein level. The significance calls for each sample and level for the moderate significance values are displayed as a complex upset plot (Lex *et al.* 2014, Krassowski *et al.* 2022) in Fig. 4A: probe level, 4B: epitope level, and 4C: protein level. The number of probes, epitopes, and proteins recognized by 4, 5 or 6 of the 6 immune mice was substantially lower than the number of epitopes mutually recognized by multiple individuals. Supplementary Figure S5 presents data for the LEM containing protein 3 (Lemd3), illustrating where two epitopes were found in two different positive samples (1 out of 6, ∼16%), which resulted in a K of N of 2/6 (33%) at the protein level. Supplementary Figure S6 depicts the Hemicentin-1 (Hmcn1) protein, which had 11 epitopes called in different regions with different numbers of positive samples called.

### 3.4 Validation by ELISA

For the Melanoma dataset 14 of the peptides selected by HERON were further tested using ELISA. Using an internal validation cohort, 71% (10/14) of the positively selected peptides validated for positive reactivity and for the external cohort, 57% (8/14) of the positively selected peptides validated for positive reactivity. Further details are given in Supplementary Section S1 with Supplementary Fig. S7.

## 4 Discussion

Ultra-dense linear peptide binding arrays are useful for identifying the landscape of antibody binding to entire microbe or mouse proteomes for immune studies, however, many factors determine the interpretation of immune response in the form of antibody binding to linear peptides from peptide arrays. Defining antibody binding epitope boundaries can be challenging as regions of interest can be defined as regions with similar antibody binding responses across multiple samples, or a region from an individual or a small number of samples. We developed HERON, a software package that can make antibody binding calls and estimate significance at the peptide, epitope, and protein level, while identifying global and individual features.

In the COVID-19 comparison between HERON and the method described in Heffron *et al.* (2021), we found good agreement between the probe- and protein- levels found by HERON; the differences between called probes are due to (i) relaxation for every COVID+ sample that gets called separately using the standard deviation from the COVID- samples, (ii) a strict 2-fold change cutoff was used by Heffron *et al.*, whereas HERON used a less stringent method that incorporates the 2-fold change as part of the differential *P*-value calculation. Looking at the probes, we found that of the 7 missed by HERON, 4 narrowly missed the 25% cutoff (9 out of 40 COVID+ samples). The significant disagreements at the epitope level are due to differences in the way the epitopes are found and scored. The HERON method finds epitopes with a procedure that tries to balance an individual sample and across-sample levels and reports %Called scores to allow the user to determine the number of samples that are needed for valid epitopes. In contrast, the *t*-test method presented in the Heffron *et al.* (2021) study is looking for a difference of means between the COVID negative and positive samples. Looking at the epitope annotations for the spike protein of SARS-CoV-2 depicted in Fig. 3B, some of the epitope boundary differences between the Heffron *et al.* (2021)*t*-test method (Heffron *et al.* 2021) and HERON method are probably due to some samples with a high post score. The *t*-test method makes calls based upon the average across all post samples, where a few outliers from the mean could result in a higher overall mean. The HERON method makes a call on each post sample, which could separate high binding “outliers” from lower binding “inliers.” The HERON method can also find probe hits similar to pepStat, which is a well-documented and tested software toolkit for analyzing peptide array data. Most of the differences between pepStat and HERON can be attributed to edge effects, where an uncalled probe lies at the ends of epitopes that were previously found (Heffron *et al.* 2021), indicating that HERON can find epitopes that are more specific to a subset of subjects, and warrants additional study to further improve the epitope segmentation process.

As mentioned previously pepStat is designed to call peptide sequences from multiple variants of the same protein and uses a nonparametric approach to calculate FDR from the subtracted results of negative and positive samples whereas HERON uses a parametric approach.

While not currently maintained, pepBayes was shown to be superior to pepStat. On the COVID-19 dataset, HERON finds a superset of the calls found by pepBayes. Furthermore, pepBayes required more computational effort than HERON to find significantly bound probes. On the same computer (48 core AMD, 156G RAM, 2.295 GHz), pepBayes took 15.6 h to complete with 16 cores, whereas HERON, using the unpaired *t*-test, required just 4 s with one core. The less computational time required by HERON further demonstrates its utility for finding significant differentially bound probes, epitopes, and proteins.

What parameters to use depends upon the type of question and goals of the study. In the case of virus biomarker discovery, finding consistent epitopes across positive samples is desired to find a general biomarker for the development of vaccines and diagnostics. HERON also provides the ability to find positive sample-specific epitopes and for finding epitopes of longer span using the “unique” segmentation method. Finding longer epitopes may be of interest to researchers when trying to find positive sample-specific epitopes.

Our results using different meta *P*-values on the Melanoma dataset give an idea of what method to use when calculating an aggregate *P*-value for the epitope and protein level calls. Different methods need to be employed depending upon whether the desired result is for all *P*-values within the aggregate to be significant (epitope level) or at least one of the *P*-values within the aggregate need to be significant (protein level). We have chosen a few different meta *P*-value methods for comparison. In the literature, there are many other meta *P*-value methods (Kost and McDermott 2002, Poole *et al.* 2016, Sun and Lin 2020) and a future comparison of those methods with the ones studied could be warranted.

Based on our results, the Fisher method is competitive and the extensions to that method such as the empirical Brown (Poole *et al.* 2016) method that use covariance between samples to estimate *P*-values could be helpful in finding consistent probes, epitopes, or proteins across all samples when the number of samples is high. As the antibody repertoire of mice shows stochastic variability between mice, based on VDJ recombination of genes determining antigen binding immunoglobulin regions during immune ontogeny, the number of probes, epitopes and proteins expected to be recognized by serum from all immune mice is expected to be small. Our initial results evaluating this, using the methods developed herein, confirms this prediction (Hoefges *et al.* 2023). However, we have found that there are some probes, epitopes, and proteins co-recognized by a substantial fraction of immune mice, suggesting some antigens may be of importance in a substantial fraction of mice. For epitope meta *P*-values, we use methods that ensure that the underlying *P*-values are mostly significant for epitope scoring. The one-hit filter seems to increase the correlation results slightly at the cost of reduced number of hits. Although the hits that were missed may be due to noise from the peptide array.

There are several complexities with running HERON for processing peptide binding array datasets. Starting at the unique sequence level, HERON assumes that the data is normally distributed, or if the data has been smoothed, the smoothed probe level is normally distributed. Supplementary Section S2 discusses the known assumptions and possible ways to address or relax them.

In conclusion, HERON, an R package for analyzing peptide binding array data, is a flexible and powerful tool for selecting groups of linear peptide probes with improved reliability and reproducibility when considering epitopes rather than single peptide probes due to several factors. First, there are many more probes than epitopes in the proteome, giving a larger number of possible mismatches. Second, an individual epitope can be a component of several overlapping probes; our algorithm for detecting epitopes recognized by separate assessments of serum samples, requires a degree of similar recognition of the related epitope containing probes by at least 2 samples, but does not require complete identity of probe recognition and signal. This enables higher reproducibility of epitopes recognized with high signals rather than just peptides recognized at high signals when replicate chips are evaluated for separate aliquots of the same immune serum sample and when evaluating proteins that are recognized, since a single protein might be recognized by different individuals at different regions.

## Supplementary Material

btae637_Supplementary_Data

## Supplementary data

Conflict of interest: S.J.M. and I.M.O are listed as the inventors on a patent filed that is related to findings in the COVID manuscript. Application: 63/080568, 63/083671. Title: IDENTIFICATION OF SARS-COV-2 EPITOPES DISCRIMINATING COVID-19 INFECTION FROM CONTROL AND METHODS OF USE. Application type: Provisional. Status: Filed. Country: United States. Filing date: 18 September 2020, 25 September 2020. Other than these affiliations, the authors declare that the research was conducted in the absence of any commercial or financial relationships that could be construed as a potential conflict of interest.
