# Supplementary material for: Ranking antibody binding epitopes and proteins across samples from whole proteome tiled linear peptides
Source: Bioinformatics. 2024 Nov 5;40(12):btae637. doi: 10.1093/bioinformatics/btae637 (PMC11631460; doi:10.1093/bioinformatics/btae637)
Supplement: btae637_Supplementary_Data [file btae637_supplementary_data.zip › HERON_Bioinf_2024_resubmit_1_Supplement_clean.docx]

Ranking Antibody Binding Epitopes and Proteins Across Samples from Whole Proteome Tiled Linear Peptides

Sean J. McIlwain1,2,*, Anna Hoefges3, Amy K. Erbe3, Paul M. Sondel2,3,4, and Irene M. Ong1,2,5,6,*

1Department of Biostatistics and Medical Informatics, University of Wisconsin-Madison, WI, 2University of Wisconsin Carbone Comprehensive Cancer Center, University of Wisconsin-Madison, WI 3Department of Human Oncology, University of Wisconsin-Madison, WI, 4Department of Pediatrics, University of Wisconsin-Madison, WI, 6Department of Obstetrics and Gynecology, University of Wisconsin-Madison, WI, 6Center for Human Genomics and Precision Medicine, University of Wisconsin-Madison, WI

*To whom correspondence should be addressed.

E-mail: [sean.mcilwain@wisc.edu](mailto:sean.mcilwain@wisc.edu), [irene.ong@wisc.edu](mailto:irene.ong@wisc.edu)

Supplementary Material

Table of Contents

[S1. Validation by ELISA 3](#_Toc160701064)

[S2. Assumptions, Issues, and Calibration 3](#_Toc160701065)

[S2.1 Assumptions on the unique sequence or smoothed probe-level 3](#_Toc160701066)

[S2.2 Assumptions on the epitope-level 4](#_Toc160701067)

[S2.3 Assumptions on the protein-level 4](#_Toc160701068)

[S2.4 Calibration of p-values using permutation tests 4](#_Toc160701069)

[S3. Supplementary figures 5](#_Toc160701070)

[Figure S1 - Probe estimated p-values vs normalized signal for one representative serum sample from an immune mouse (AC5) included in the melanoma dataset using moderate statistics parameters. 5](#_Toc160701071)

[Figure S2 – Probe-level Performance Comparison with different algorithms for COVID-19 Data. 6](#_Toc160701072)

[Figure S3 - Performance Results for Melanoma Dataset. 7](#_Toc160701073)

[Figure S4 - Technical replicate scatterplots of -log10 of adjusted p-values on probes, epitopes, and proteins using the moderate level statistics. 8](#_Toc160701074)

[Figure S5 - Heatmap and line plots of Lemd3. 9](#_Toc160701075)

[Figure S6 - Heatmap and Lineplots of Hmcn1. 10](#_Toc160701076)

[Figure S7 - Heatmaps of ELISA results. 11](#_Toc160701077)

[References 12](#_Toc160701078)

# S1. Validation by ELISA

For the Melanoma dataset, the probe, epitope, and protein calls using the one-hit filter, unique segmentation method, the Wilkinson’s max for epitopes, and min+bonf for proteins with the inclusive, moderate, and restrictive significance levels were used to select peptides for validation. Hoefges et al 2023 selected 16 peptides (16-mers that had been tested in the high-density array) to validate using ELISA; 14 were selected based on their strong signal > 6SD over the mean by at least 3 of the 6 immune serum samples tested in the high-density array. The other 2 were selected as negative controls, based on signals < 3SD over the mean for all 6 immune serum samples in the high-density array. These were all then tested in a standard ELISA assay, with data reported out as optical density (O.D.) values for each serum sample (naïve or immune) tested, as detailed by Hoefges et al 2023 (Hoefges, et al., 2023).

The replicates for ELISA were first averaged together. To indicate a positive hit, a threshold O.D. value of greater than or equal to two was used on each ELISA data point. For each peptide, the fraction of positive hits was calculated for the immune samples in the original and validated ELISA set and for the pre-/Naive samples in the validated set. A peptide was called validated for positive reactivity if 25% or more of the respective pre-/Naïve or post-/Immune samples were called.

The Internal Validation Cohort consisted of immune and naïve sera from the original 6 immune mice used in the original high-density array (and shown in Figure 5). When these sera were tested in the ELISA on these 16 peptides (**Supplementary Fig. S6A**), 10 out of 14 positively-selected peptides (71%) validated for positive reactivity on the immune samples, while 0 (0%) of the 14 peptides validated for positive reactivity on the naïve samples, as expected. In contrast, 0 out of 2 negatively-selected peptides (0%) had validated for positive reactivity on the immune samples, and similarly 0 out of 2 negatively-selected peptides (0%) had positive reactivity on the pre-/naive samples, as expected.

The External Validation Cohort consisted of serum samples from 20 separate immune mice and naïve serum samples from 14 of those mice, that had not ever been tested before in any high-density array or any ELISA assay. When these 34 sera were tested in the ELISA on these 16 peptides (**Supplementary Fig. S7B**), most (8 out of 14) positively-selected peptides (57%) validated for positive reactivity on the immune samples, as expected, while few (only 2 of the 14) peptides (14%) had positive reactivity on the naïve samples, as expected. In contrast, 0 out of 2 negatively-selected peptides (0%) validated for positive reactivity on the immune samples, and similarly 0 out of 2 negatively-selected peptides (0%) validated for positive reactivity on the pre-/naive samples, as expected. These results with this external, independent, validation cohort are showing a similar, but not quite identical, pattern as seen with the internal validation cohort; the only difference is some of the naïve sera of these 14 independent cohort mice tested were also detecting a fraction of the positive peptides, which was not seen with naïve sera from the original cohort of 6 immune mice. The naïve sera from the original 6 mice did recognize some peptides in the high density array, but these peptides were not chosen for this validation ELISA testing. Thus, it is not surprising that some of the naïve sera in the independent cohort of mice might be able to recognize some peptides not recognized by the naive sera of the original cohort (**Supplementary Fig. S6**).

# S2. Assumptions, Issues, and Calibration

## S2.1 Assumptions on the unique sequence or smoothed probe-level

For calculating the differential t-test p-values/scores, HERON assumes that the variance of the post-sample is the same as the estimated variance of the pre-sample and that the post-sample signal represents the mean of the post-group values. To help deal with the higher variance and the obvious breakdown of the assumptions, we use the degrees of freedom from the pre-samples to estimate the one-sided p-value from the t-distribution.

Upon calculating the global z-test p-values/scores, HERON assumes that the sequence or smoothed probes signals are comparable across different sequences and that the mean and standard deviations can be used to calculate the p-value against one sequence signal for a post sample.

When combining the differential and global z-test p-values, the Wilkinson’s max meta p-value method assumes independence among the p-values to be combined. More study is needed to determine if HERON has violated this assumption and, if so, the ramifications when estimating the combined p-values/scores or if using a different meta p-value that more tolerant to correlated p-values would improve HERON’s estimation of significance on the probe-level.

Finally, in the case of copying the unique sequences to the probe-level, there is an assumption that it is fair to do so. The main assumption is that resulting probe-level p-values are still well behaved. In the case of the Melanoma dataset, since we are smoothing the data beforehand, each probe can be treated as an independent measurement, even though there are some peptide probes that come from a sequence that maps to more than one protein. The COVID-19 dataset, however, is unsmoothed and contains many sequences shared between proteins from different strains of the coronaviruses. To process unsmoothed data in HERON, we estimate the adjusted p-values on the unique sequences and then copy the result to the respective probe-level identifiers.

## S2.2 Assumptions on the epitope-level

For the epitopes, or group of consecutive probes across a protein, the meta p-value methods used assume that the p-values are accurate and well-calibrated. Several of them (Wilkinson, min+bonf, Fisher, etc.) assume independence among the p-values, which is directly violated as we group adjacent probes which overlap, as epitopes. Other methods such as the Cauchy combination test or the harmonic mean try to alleviate this assumption, while other meta p-value methods (Brown, Kosts, etc.) attempt to model the covariance between the p-values to improve the estimation.

There is also an assumption that the epitopes identified by the epitope finding methods are the only ones to be included in the analyses. For example, do we need to correct for the number of all possible epitopes per protein? Currently, HERON treats the list of epitopes as a separate list and just corrects using the Bonferroni-Hochberg algorithm when reporting the adjusted p-values for the epitopes and uses the uncorrected p-values for estimating the protein-level p-values.

## S2.3 Assumptions on the protein-level

Going up another level in the hierarchy of probes, epitopes, and proteins, the protein p-values calculations assume that the epitope regions and p-values/scores are well-calibrated and well-behaved and the further assumptions made by the meta p-value method used to estimate the protein p-value/scores from the epitope p-values. We also assume that the adjusted p-value correction only needs to be applied to the proteins for which at least one epitope region was found.

## S2.4 Calibration of p-values using permutation tests

One of the ways to alleviate some of the issues presented above is to re-calibrate the scores into actual accurate/well-behaved p-values. Permutation statistics, shuffling the sample labels, calculating the resulting p-values/score, and using the permutation scores to calibrate the raw p-values is a popular method for ensuring well behaved statistics.

While the use of permutation statistics to provide accurate p-values on the unique sequence or probe-level is seemingly straightforward (Data not Shown), it is unclear how to properly perform this calibration at the epitope, and protein levels. We are currently exploring this avenue to see if providing calibrated p-values to the unique/smoothed probe-level also equivalently gives well calibrated scores at the epitope and protein level, or if more complicated calibration is needed.

Furthermore, permutation statistics is also affected by the number of samples used in the experiment. For example, in the Melanoma experiment, there are 8,459,970 unique peptide probes (not 6,090,593 due to the smoothing across probes), and only 11 biological samples. Testing for all possible permutations (11! = 39,916,800) would achieve a minimum permutation estimated p-value of 2.505x10-8, which after using a Bonferroni correction against all probes would achieve a corrected p-value (2.505x10-8 x 8,459,970) of 0.212 or 0.153 using 6,090,593 unique sequence probes. While there are methods to further increase the p-value accuracy in the lower range of p-values (Knijnenburg, et al., 2009) with fewer permutations, there is a limitation with using permutation tests with millions of features and a small set of independent biological samples.

Finally, recent studies have shown that care must be taken when using the permutation test techniques (Christensen and Zabriskie, 2022). In this paper, we use the normalized log-transformed data, which is part of the suggested use of the Box-Cox transformation (Box and Cox, 1964). Other methods for analyzing high-throughput peptide binding array data perform a log-log transformation before estimating the statistics (Mergaert, et al., 2022; Zheng, et al., 2021). While studying the application of permutation calibration, we will investigate the usefulness of the Box-Cox transformations when calculating the statistics and calibration procedures.

# S3. Supplementary figures

**Figure S1 - Probe estimated p-values vs normalized signal for one representative serum sample from an immune mouse (AC5) included in the melanoma dataset using moderate statistics parameters**. In each panel, each dot represents the value obtained for this single serum sample on the ~8x106 peptide probes (16-amino acids each) tested in the Nimble peptide array system. A) result of the differential p-value estimation using t-test. B) result of global p-value using z-test. C) result of the combined p-value using Wilkinson’s max. D) result of adjusted p-values (Benjamini-Hochberg).

**Figure S2 – Probe-level Performance Comparison with different algorithms for COVID-19 Data.** Upset plot of the probe-level calls using HERON, HERON using Wilcoxson with exact p-value tests, pepStat, and pepBayes. Calls were made using an FDR/padj threshold of 0.01 and a %Called to be at least 25%. Calls from (Heffron, et al., 2021) included for comparison.

**Figure S3 - Performance Results for Melanoma Dataset. (A)** Average Correlation between technical replicates for the Top 10 algorithm parameter settings. Each row-label is as follows: One hit filter (T - True or F – False indicates whether filter is used or not), Segmentation method (*uniq* – unique set of epitopes found across all post samples, *hbh* - hierarchical clustering with binary calls with hamming distance, *sbh* - skater with binary calls and hamming distance, and *sbz* - skater with z-score and Euclidean distance), epitope meta p-value method (*wmax2* – Wilkinson's max on the 2nd largest p-value, *fisher* – Fisher's method), protein meta p-value method (*tippetts* – Tippett's or Wilkinsons’s min on the 1st smallest p-value, *min_bonf* – finds the minimum p-value then correct by the number of epitopes on the proteins using Bonferroni), *wmin2* – Wilkinson min on the 2nd smallest p-value). (B) Average Epitope Sequence Length vs. Segmentation Method. Box of the average sequence length that results from stitching the contained probe sequences for the detected epitope. For each segmentation method, the box is calculated using all of the average epitope sequence lengths for the remaining parameters, keep the epitopes that have at least one sample called.

**Figure S4 - Technical replicate scatterplots of -log10 of adjusted p-values on probes, epitopes, and proteins using the moderate level statistics.** A, B, and C are from the technical replicates collected on the same array (two separate replicate data sets obtained in the same assay using split serum aliquots from immune mouse B2). D, E, and F are from the technical replicates collected on arrays collected at different times (two separate replicate data sets obtained on replicate high density array chips in assays performed one-year apart, using split serum aliquots from immune mouse PD1). A and D are from the probe-level p-values, B and E are from the epitope-level p-values, and C and F are from the protein-level p-values. Light-blue are the probes, epitopes, or proteins that are called in just the 1st replicate shown on the X-axis (B2-1 or PD-1). Dark blue are the probes, epitopes, or proteins that are called in the 2nd replicate (B2-2 or PD-2) shown on the Y-axis. Medium blue are the probes, epitope, or probes that are called in both replicates (and thus running more on the diagonal). For A and D, the black points indicate probes that were not called in either replicate.

**Figure S5 - Heatmap and line plots of Lemd3.** (A) Line charts and Heatmap of Lemd3 using normalized and smoothed intensity values. The X-axis indicates the starting position of the probe within the protein and the Y-axis rows consist of the individual pre-treatment (Naïve) and post-treatment (Immune) samples. The first line chart above the heatmap indicates the percent of positive samples that were called on the epitope-level (Moderate Significance), the second line chart indicates the percent of positive samples called at the probe-level (Moderate Significance), and the third line chart shows the average signal between negative (Naïve) and positive (Immune) samples. (B) Line plot of epitope detected for the mouse (AC5) sample. (C) Line plot of the epitope detected in the mouse (C4) sample. For both (B) and (C), the Y-axis is the normalized intensity values and the X-axis is the starting position of the probe within the protein and the dark green dotted lines indicate the samples that were called within the epitope boundary, which are indicated by vertical black or orange lines. The orange line indicates a probe tested and validated by ELISA.

**Figure S6 - Heatmap and Lineplots of Hmcn1.** (A) Line charts and Heatmap of Lemd3 using normalized and smoothed intensity values. The X-axis of indicates the starting position of the probe within the protein and the Y-axis is the individual pre- and post- samples. The first line chart above the heatmap indicates the percent of positive samples that were called on the epitope-level (Moderate Significance), the second line chart indicates the percent of positive samples called at the probe-level (Moderate Significance), and the third line chart shows the average signal between negative (Naïve) and positive (Immune) samples. (B) Line plots of 11 epitopes detected for the various mouse samples, Y-axis is the normalized intensity values and the X-axis is the starting position of the probe within the protein. The dark green dotted lines indicate the samples that were called within the epitope boundary, which are indicated by vertical black or orange lines. The orange line indicates a probe tested and validated by ELISA.

**Figure S7 - Heatmaps of ELISA results**. Sixteen-mer peptides tested are shown on the columns and the individual naïve and immune serum samples are indicated on the rows. The top bar plots indicate the percentage of calls made on the probe-level by HERON in the Inclusive, Moderate, and Restrictive significance levels based on the original high density peptide array data (note these data are shown for comparison, and the graph of these high-density data are replicated for comparison’s sake in the top panels for A and B). The lower bar plot indicates the percentage of Naïve (grey) and Immune samples (green) that had an average ELISA value ≥ 2 O.D. intensity. Below these are the heat maps, showing the average across replicates O.D. values for each serum sample (naïve or immune) tested against each of the 16 peptides, with the 2 negative peptides shown at the far left and the 14 positive peptides at the right. (A) Internal Validation Cohort, (B) External Validation Cohort. Missing values (due to insufficient volume of serum available for that sample) are indicated as black values in the heatmaps.

# References

Box, G.E.P. and Cox, D.R. An Analysis of Transformations. *Journal of the Royal Statistical Society: Series B (Methodological)* 1964;26(2):211-243.

Christensen, W.F. and Zabriskie, B.N. When Your Permutation Test is Doomed to Fail. *The American Statistician* 2022;76(1):53-63.

Heffron, A.S.*, et al.* The landscape of antibody binding in SARS-CoV-2 infection. *PLoS Biol* 2021;19(6):e3001265.

Hoefges, A.*, et al.* Antibody landscape of C57BL/6 mice cured of B78 melanoma via a combined radiation and immunocytokine immunotherapy regimen. *Front Immunol* 2023;14:1221155.

Knijnenburg, T.A.*, et al.* Fewer permutations, more accurate P-values. *Bioinformatics* 2009;25(12):i161-168.

Mergaert, A.M.*, et al.* Rheumatoid Factor and Anti–Modified Protein Antibody Reactivities Converge on IgG Epitopes. *Arthritis & Rheumatology* 2022;74(6):984-991.

Zheng, Z.*, et al.* MixTwice: large-scale hypothesis testing for peptide arrays by variance mixing. *Bioinformatics* 2021.
