# Supplementary figures and images for: Ranking antibody binding epitopes and proteins across samples from whole proteome tiled linear peptides
Source: Bioinformatics. 2024 Nov 5;40(12):btae637. doi: 10.1093/bioinformatics/btae637 (PMC11631460; doi:10.1093/bioinformatics/btae637)

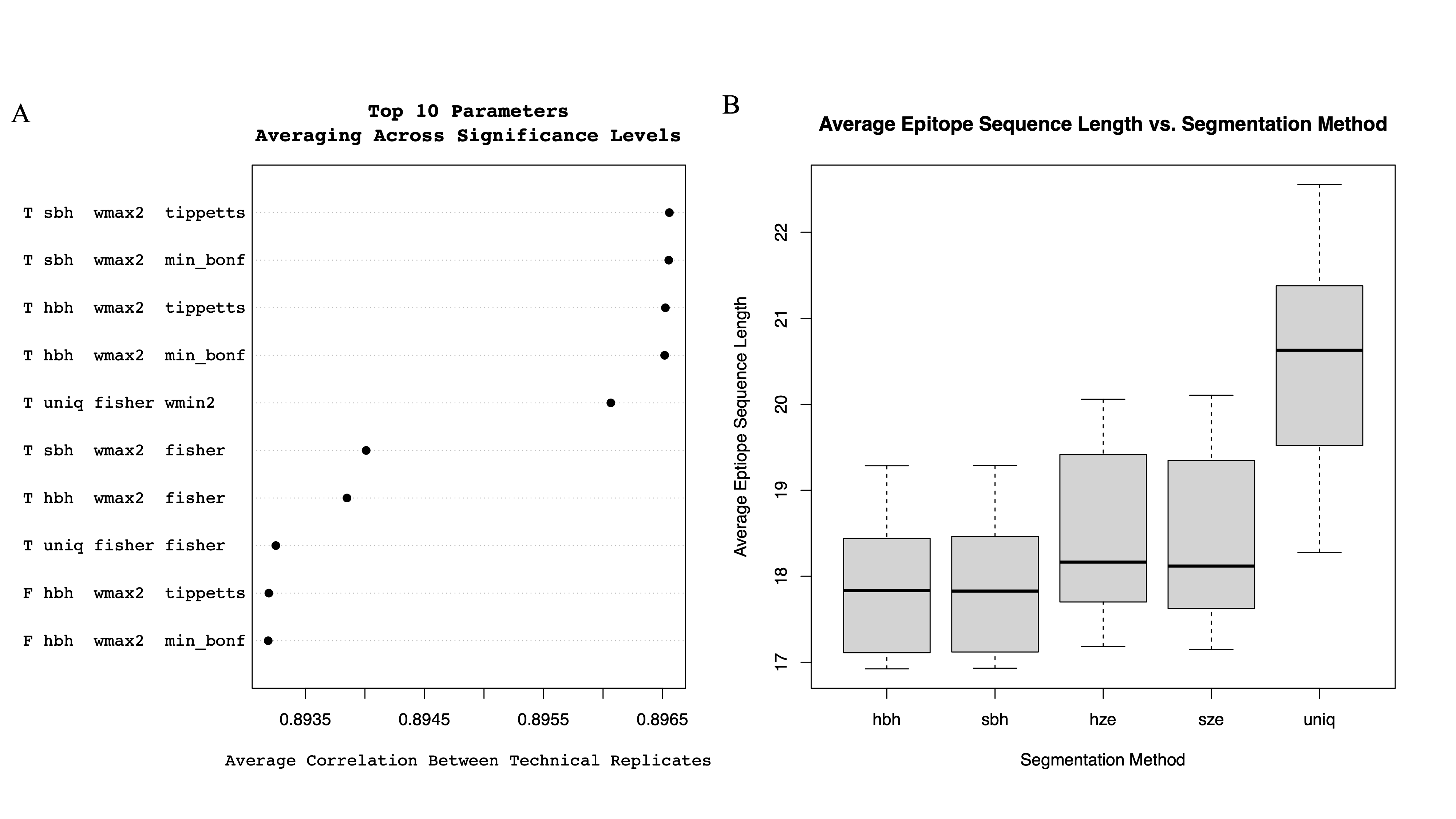

Supplement: btae637_Supplementary_Data [file btae637_supplementary_data.zip › SFig3.png]

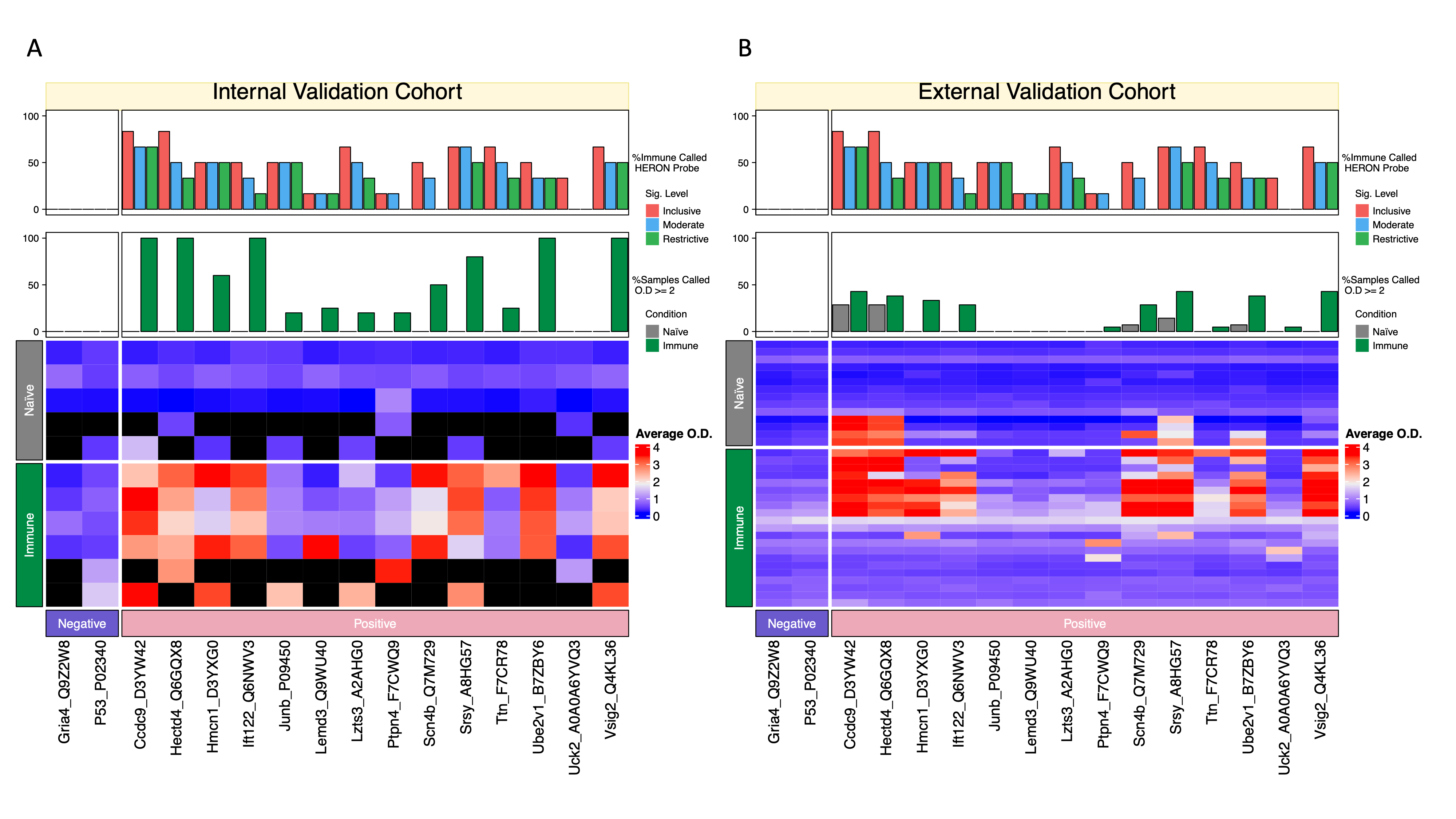

Supplement: btae637_Supplementary_Data [file btae637_supplementary_data.zip › SFig7.png]

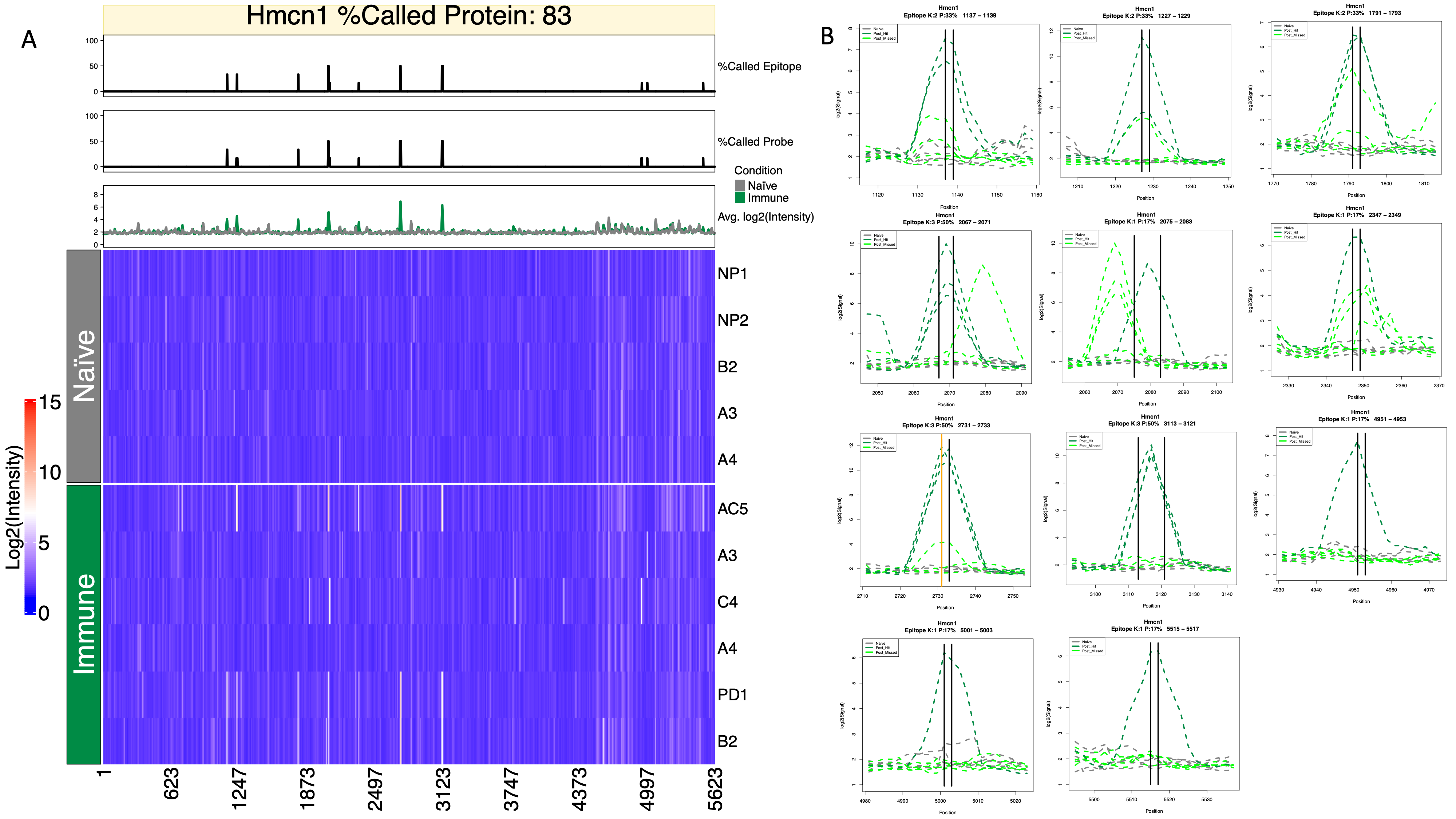

Supplement: btae637_Supplementary_Data [file btae637_supplementary_data.zip › SFig6.png]

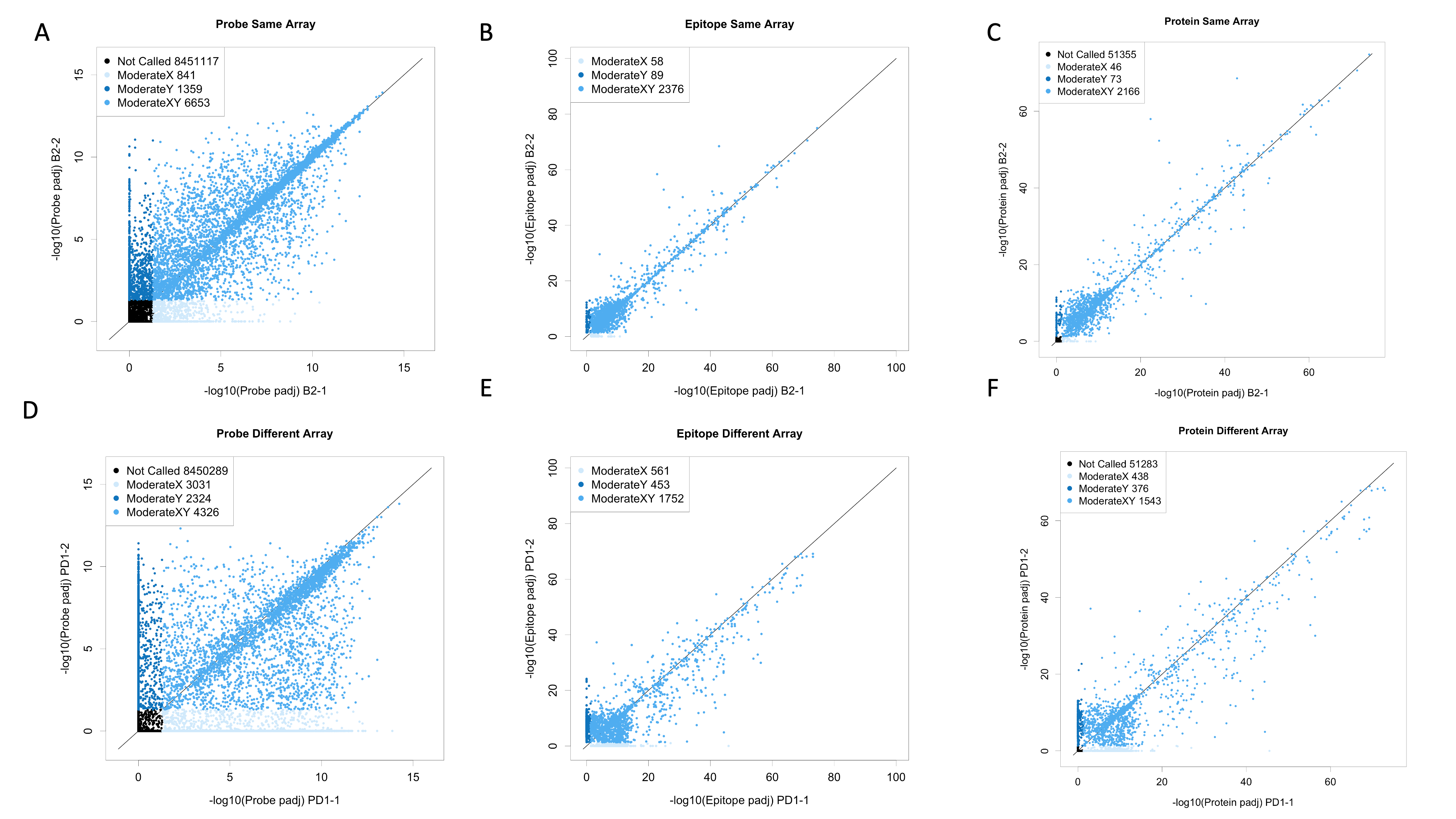

Supplement: btae637_Supplementary_Data [file btae637_supplementary_data.zip › SFig4.png]

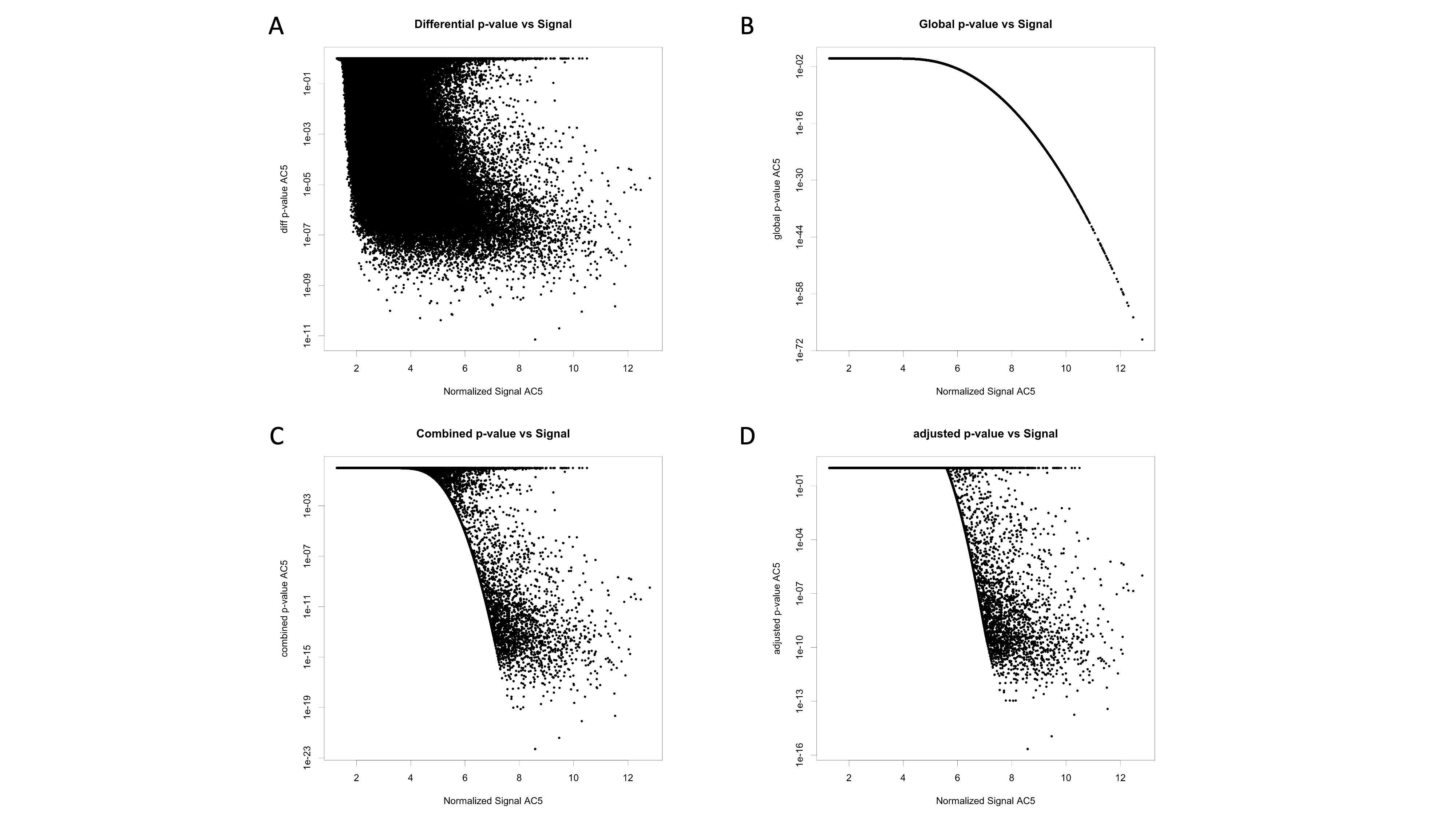

Supplement: btae637_Supplementary_Data [file btae637_supplementary_data.zip › SFig1.png]

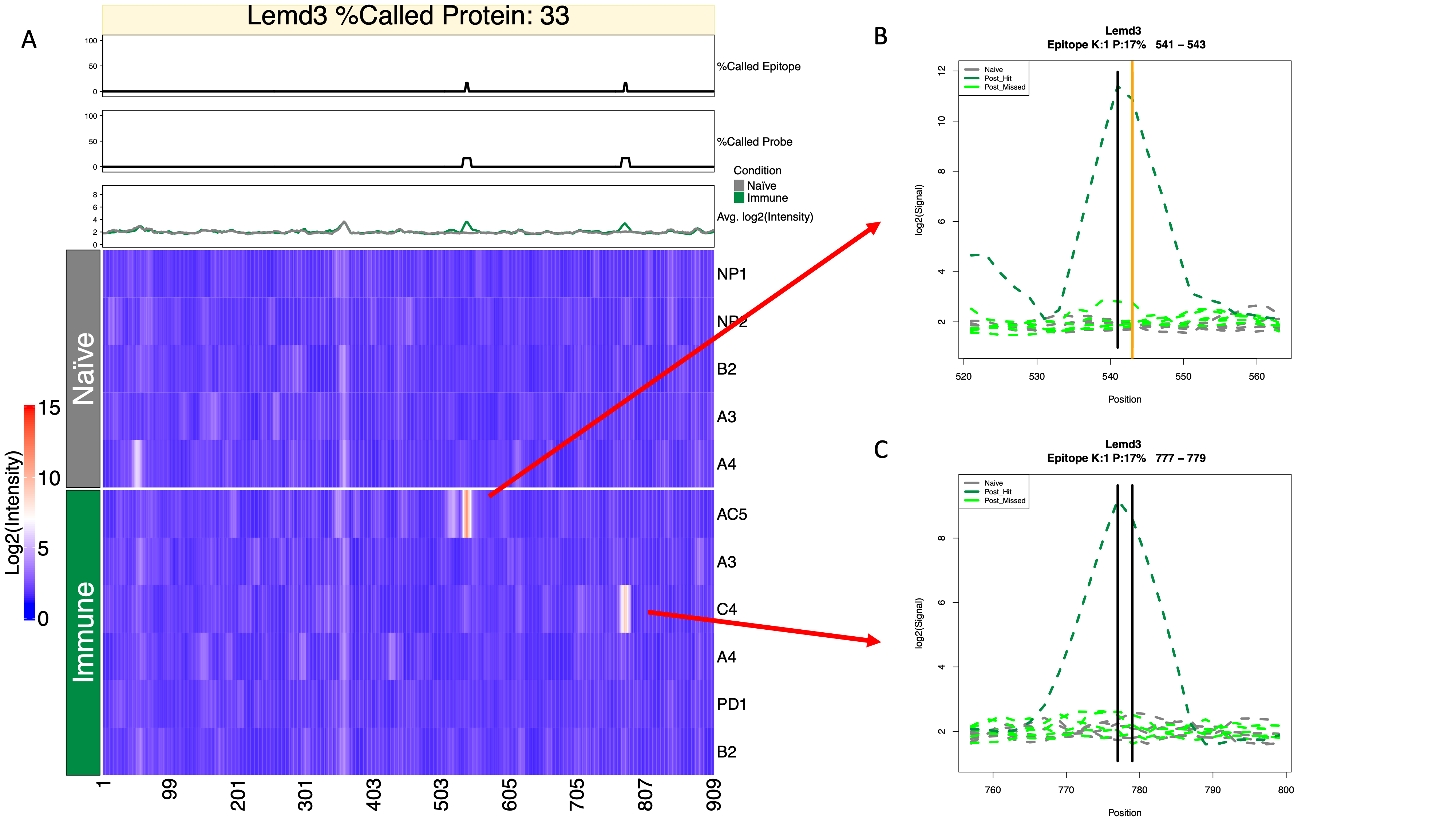

Supplement: btae637_Supplementary_Data [file btae637_supplementary_data.zip › SFig5.png]

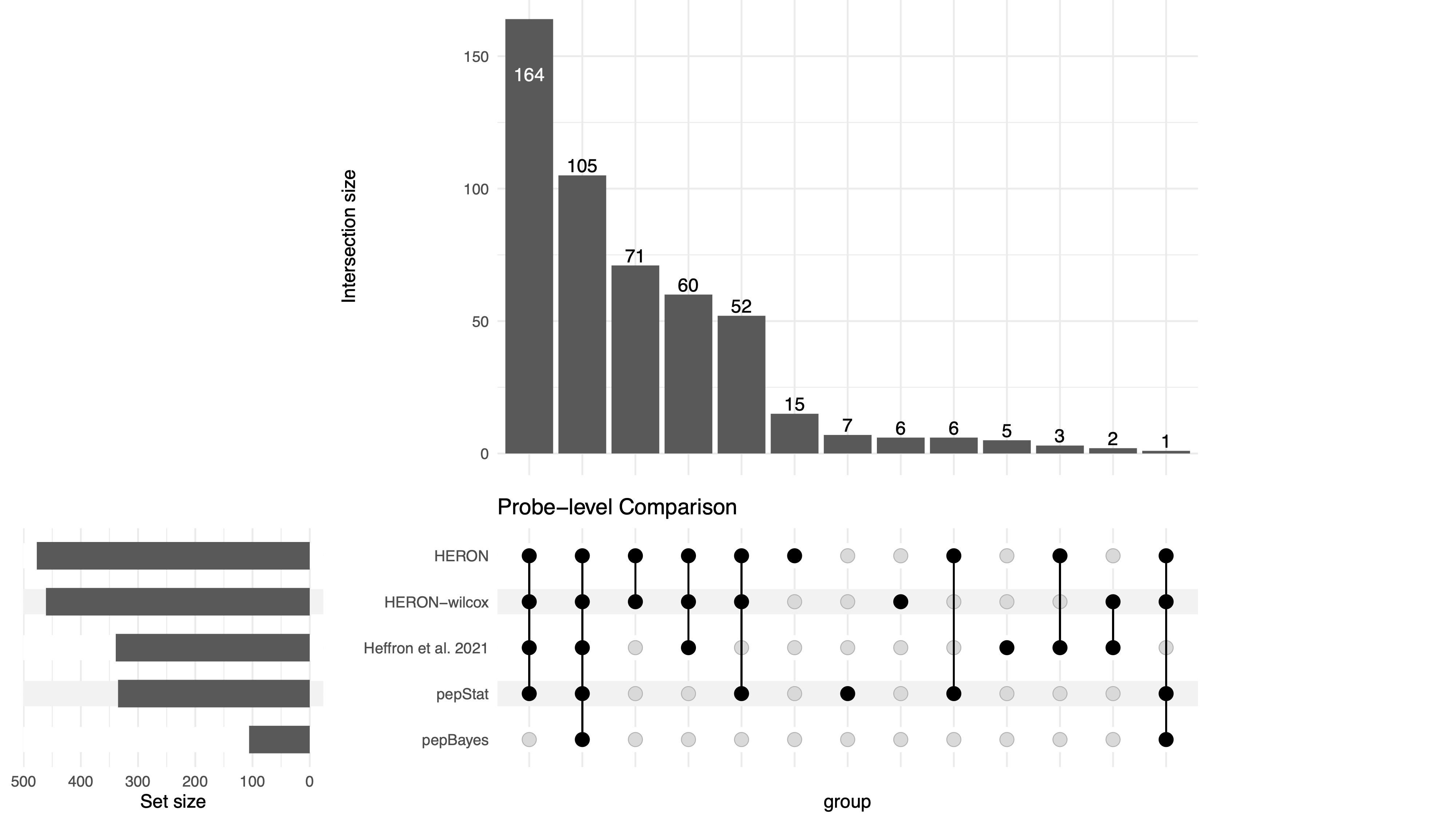

Supplement: btae637_Supplementary_Data [file btae637_supplementary_data.zip › SFig2.png]
